# Prevalence of and Factors Associated With Nutritional Supplement Use Among Older Chinese Adults: A Nationwide Cross-Sectional Study in China

**DOI:** 10.3389/fpubh.2022.822087

**Published:** 2022-03-24

**Authors:** Wanyue Dong, Zhonghe Sun, Ruhai Bai

**Affiliations:** ^1^School of Elderly Care Services and Management, Nanjing University of Chinese Medicine, Nanjing, China; ^2^Department of Social Work, Nanjing First Hospital, Nanjing Medical University, Nanjing, China; ^3^School of Public Affairs, Nanjing University of Science and Technology, Nanjing, China

**Keywords:** nutritional supplement use, older adults, prevalence, associated factors, China

## Abstract

**Objective:**

This study identified the prevalence of nutritional supplement (NS) use among older Chinese adults and explored the factors associated with NS use in this population.

**Methods:**

We used data from 11,089 Chinese men and women aged ≥ 65 years from the 2018 Chinese Longitudinal Healthy Longevity Survey. The chi-square test was used to examine the differences in demographics, health status and lifestyles at different levels. Multivariate logistic regression was used to assess the association between NS use and demographic and lifestyle characteristics.

**Results:**

Twelve percent of Chinese adults aged 65 years and above used NS. In terms of the type of supplement used, the most commonly used was calcium (8.49%), followed by protein (2.73%) and multivitamins (2.40%). In terms of demographic characteristics, women, older people, urban residents with other marital status, higher educational level, better living conditions and better lifestyle habits showed a greater use of some kinds of NS to varying degrees. Factors associated with the use of any NS included female gender [OR = 1.71, 95% confidence intervals (95% CI): 1.09–1.44], age 85–94 (OR = 1.30, 95% CI: 1.08–1.58), urban household registration (*hukou*) (OR = 1.25, 95% CI:1.46–2.00), higher education (primary school and middle school: OR = 1.32, 95% CI:1.14–1.52; high school and above: OR = 1.56, 95% CI:1.25–1.94), average and poor living standard (average: OR = 0.64, 95% CI:0.56–0.73; poor: OR = 0.42, 95% CI:0.32–0.55), poor health status (OR = 1.36, 95% CI:1.13–1.63), former smoking (OR = 1.33, 95% CI:1.11–1.60), and having exercise habits (former exercise: OR = 2.24, 95% CI:1.83–2.74; current exercise: OR = 2.28, 95% CI:2.00–2.61). Women reported taking 2–3 kinds of NSs, and more than 50% of NS users reported taking supplements often.

**Conclusion:**

This study provides information on the current prevalence of NS use among older Chinese adults, and it clarifies the association of NS use with demographic, lifestyle and other factors. Providing scientifically based health guidance on NS use for older people is crucial to promoting their health.

## Introduction

Due to economic development and changing lifestyles, individuals are increasingly interested in health, wellbeing and self-care (1), especially older people who have greater health care needs and a greater concern for their health. A specific manifestation is their more widespread use of dietary supplements (2). A study of nutritional supplement (NS) use in China from 2010 to 2012 showed that participants aged 60 years and older had the highest rate of NS use among all age groups over 6 years (3).

Although scholars have conducted comprehensive studies on the role of NS in health, little is known about its prevalence, especially among older Chinese adults. Currently, most studies by Chinese scholars on NS use have been conducted in children and pregnant and lactating women, such as the effect of NSs on anemia in children (4) and the use of NSs in pregnant and lactating women (5, 6). Some scholars have studied the use of NSs in older adults with diseases (7), but studies on NS use in the older population are relatively scarce, and the results vary widely. A study by Gong on the use of NSs in China from 2010 to 2012 found that the rate of overall nutrient supplement use among people aged 60 years and older was 1.75% (3), while another 2017 resident survey on NS behaviors concluded that 45.6% of the elderly population had consumed NSs (8). On the one hand, the NS market in China has shown rapid growth over the past 20 years (9). In China, NSs are classified as a health food (10), meaning that supplement products, including vitamins, minerals and amino acids, can be purchased without a prescription, allowing for increased sales and industry expansion through advertising by sellers in the health care industry (11). However, such health-related promotion can also put consumers at risk, which especially affects the most vulnerable populations (12). On the other hand, as health literacy has increased (13, 14), the population has become skeptical of products that are marketed as “health buys.” The results from an interview study with older people in Chinese communities showed that many older participants did not trust the marketing of NSs and did not believe that these products would help to improve their health (15). “The Scientific Consensus on the Use of Nutrient Supplements among Chinese Residents” guides residents on the possible negative effects of excessive supplementation (16). Based on the above industry background and the health perceptions of the population, the available evidence does not yet give us an up-to-date picture of NS use in the older populations in China.

To fill the gaps in the literature, the purpose of this study was to examine NS use in the elderly population in China based on a nationwide survey of older adults and to explore the current prevalence of specific NS use and the factors associated with it.

## Methods

### Data Source

**Figure 1:**
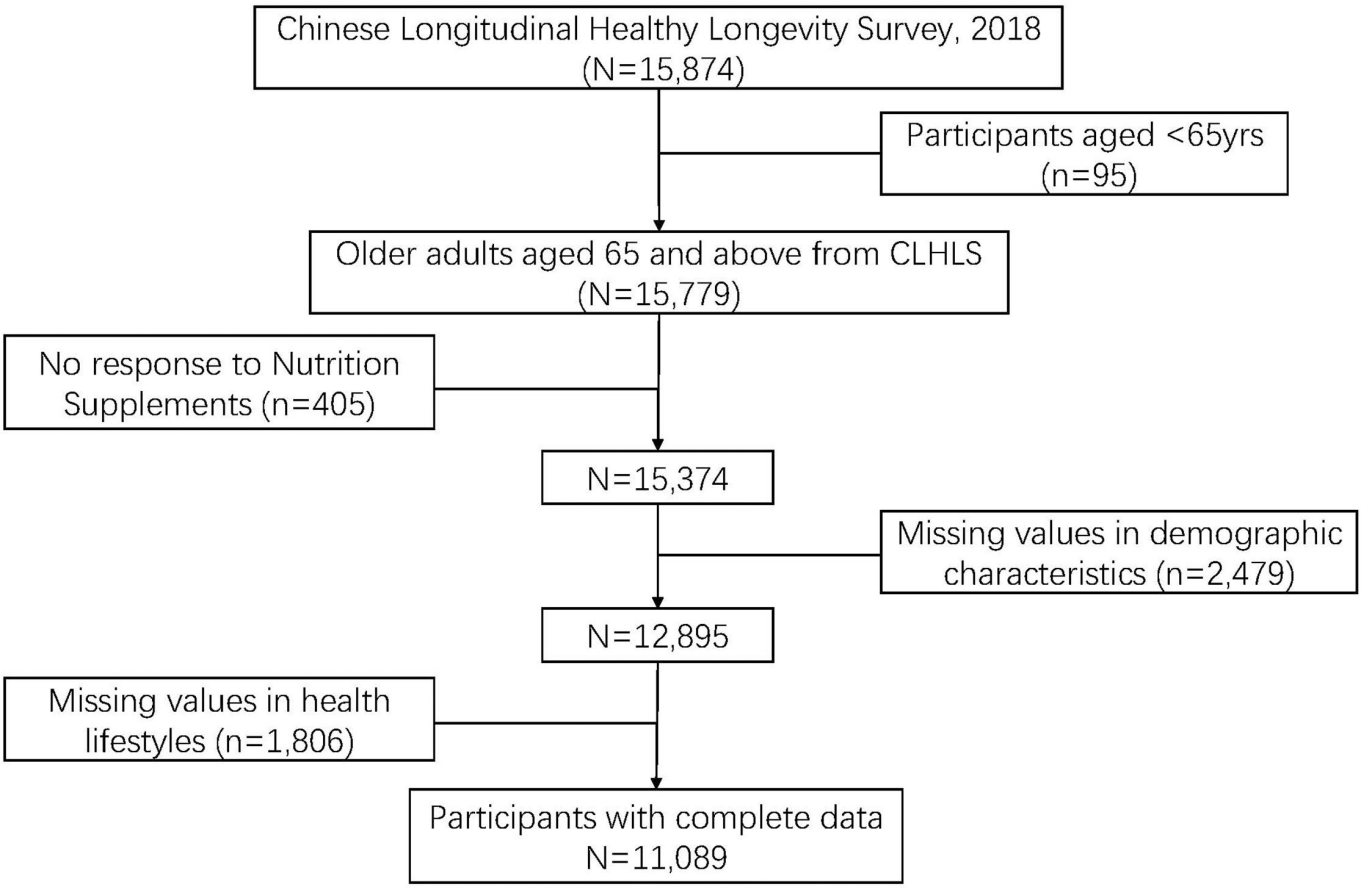
Flowchart of sample screening for study inclusion.

The data in this study were collected from the Chinese Longitudinal Healthy Longevity Survey (CLHLS), which involved a large-scale, household-based open cohort study. The CLHLS survey sampling covers ~85% of the total population in China, targeting a substantial amount of centenarians, non-agenarians, and octogenarians, with a targeted random sampling method. It was carried out in eight survey waves during the years 1998–2018 by conducting face-to-face interviews. Each respondent was interviewed by well-trained investigators. It was reported with high reliability and validity in the analyses of health measures, which exceeded widely used criteria (17). More details of the 2018 wave CLHLS data are available in the review by Zheng (18). Detailed instructions about the database are available on the website of Peking University Center for Healthy Aging and Development http://chads.nsd.pku.edu.cn/sjzx/index.htm (accessed on 10 August 2021). The data were made available through an agreement with the Center and can be downloaded from the following website: https://opendata.pku.edu.cn/dataset.xhtml?persistentId= doi: 10.18170/DVN/WBO7LK. We extracted cross-sectional data from the 2018 wave of the CLHLS because information on NS use among older people was only included in 2018 survey. A total of 15,874 respondents was obtained. Figure 1 displays the exclusion of respondents who did not meet the criteria with incomplete or missing data. A total of 11,089 participants with complete data were included in the analysis. Institutional Review Board (IRB) approval was waived, as our study used secondary data from the public domain.

### Measures

Information about nutritional supplements was collected by asking the following questions: “Do you usually take a nutritional supplement?” If the participant answered “yes,” further questions were asked, “Do you take (1) protein, (2) calcium, (3) iron, (4) zinc, (5) multivitamin, (6) vitamin A/D, (7) docosahexaenoic acid (DHA) (8) others?” and “How often do you take each kind of nutritional supplement?” Respondents answered each of nutritional supplement with a “yes” or “no” answer, followed by a selection of “seldom/ sometimes/ often” to each NS if the answer is “yes.”

All covariates used in the analysis were self-reported data obtained from face-to-face interviews. Gender was categorized as a binary variable: male or female; age was categorized as a categorical variable: 65–74, 75–84, 85–94, 95 years old and above; according to the current type of household registration (*hukou*), older adults were categorized as urban and rural, a binary variable; marital status was divided into married and others (including divorced, widowed and never married) as a binary variable; the educational level was divided into illiterate, primary school and middle school, high school and above, as a categorical variable; the living standard was based on the question “How do you rate your economic status compared with others in your local area?” This question was divided into three groups (very good/good, fair, bad/very bad, as a categorical variable). Health status was defined as the participant's self-rated health scale and was divided into three groups as a categorical variable: very good/good, fair, bad/very bad. Sleep quality was based on self-reported sleep quality (very good/good, fair, bad/very bad, as a categorical variable). The classification of smoking status, drinking status, and exercise status was based on the following questions: “Do you smoke/drink alcohol/do exercises regularly at present?” and "Did you smoke/drink alcohol/do exercises regularly in the past?” It was further divided into three groups: never, former, and current (Supplementary Table 1).

### Statistical Analysis

The usage rate of NSs was described by the rate and 95% confidence intervals (95% CI). The chi-square test was used to examine the differences in NS use in different demographics, health statuses, and lifestyles. Multivariate logistic regression was used to assess the association between NS use and demographic and lifestyle characteristics. Odds ratios (ORs) and 95% CIs were reported in the results of regression analysis. The regression results were two-tailed with a level of significance of 0.05 (*p* < 0.05). All statistical analyses were performed using STATA 14.0.

## Results

### Prevalence and Types of NSs Used by Older Adults

Table 1 shows the prevalence and types of NSs used by older adults. A total of 12.28% of the respondents reported using NSs. In terms of the type of NS used, the most commonly used was calcium (8.49%), followed by protein (2.73%) and multivitamins (2.40%); there was less than a 1% probability of iron, zinc, or DHA being used. In terms of demographic characteristics, a larger proportion of females reported using NSs (13.36% in females vs. 10.94% in males, *P* < 0.001), especially calcium (9.6% in females vs. 7.13% in males, *P* < 0.001). The proportion of older adults taking protein, iron, and multivitamins showed an overall increase with age, but the oldest age group had the lowest proportion of calcium use. A greater proportion of urban residents reported using NSs in all categories. Compared with married people, people with other marital statuses had a higher rate of protein usage (2.08% in married people vs. 3.25% in others, *P* < 0.001). The proportion of older adults taking NSs increased with educational level in all categories. Older adults with good living conditions also used more NSs. NS use in older adults was associated with health status, particularly taking calcium and zinc. Older adults with better or worse sleep quality used more NSs but did not show significant differences in specific categories. Smokeless tobacco users who had quit had the highest rate of using NSs, while current smokers had the lowest use, especially for protein, calcium, multivitamins, vitamins A and D and others. The relationship between alcohol drinking and NS use was shown for protein and iron, and again, those drinkers who had quit alcohol drinking had the highest use, and current drinkers had the lowest; for older people with past or current exercise habits, NS use was higher in all categories compared to those who never exercised.

**Table 1:** Prevalence (%, 95% confidence interval) and types of NSs used by older adults.

**Variables**		***n***	**Any NSs**	**Protein**	**Calcium**	**Iron**	**Zinc**	**Multivitamin**	**Vitamin A/D**	**DHA**	**Others**
Total		11,089	12.28 (11.68–12.91)	2.73 (2.44–3.05)	8.49 (7.98–9.03)	0.84 (0.68–1.03)	0.78 (0.62–0.96)	2.40 (2.12–2.70)	1.89 (1.65–2.16)	0.58 (0.44–0.74)	1.98 (1.73–2.26)
Gender	Male	4,953	10.94 (10.09–11.85)	2.71 (2.27–3.20)	7.13 (6.43–7.88)	0.95 (0.70–1.26)	0.83 (0.59–1.12)	2.38 (1.98–2.85)	1.94 (1.57–2.36)	0.69 (0.48–0.96)	1.92 (1.55–2.34)
	Female	6,136	13.36 (12.52–14.24)	2.75 (2.36–3.20)	9.60 (8.87–10.36)	0.75 (0.55–1.00)	0.73 (0.54–0.98)	2.41 (2.04–2.83)	1.86 (1.54–2.23)	0.49 (0.33–0.70)	2.04 (1.70–2.42)
Age,y	65–74	2,731	11.64 (10.46–12.91)	1.68 (1.24–2.24)	8.79 (7.75–9.91)	0.40 (0.20–0.72)	0.44 (0.23–0.77)	1.72 (1.27–2.28)	1.50 (1.08–2.03)	0.55 (0.31–0.90)	1.57 (1.14–2.12)
	75–84	3,034	12.52 (11.37–13.76)	2.08 (1.60–2.64)	8.83 (7.85–9.90)	0.86 (0.56–1.25)	0.76 (0.48–1.14)	2.44 (1.92–3.05)	1.94 (1.48–2.50)	0.63 (0.38–0.98)	1.94 (1.48–2.50)
	85–94	2,738	13.48 (12.22–14.81)	3.4 (2.75–4.15)	9.24 (8.18–10.39)	1.06 (0.71–1.52)	0.95 (0.06–1.39)	2.78 (2.19–3.46)	2.37 (1.84–3.02)	0.69 (0.42–1.08)	2.34 (1.80–2.98)
	≥95	2,586	11.41 (10.21–12.70)	3.91 (3.19–4.73)	7 (6.05–8.05)	1.04 (0.69–1.52)	0.97 (0.63–1.42)	2.67 (2.08–3.36)	1.74 (1.27–2.32)	0.43 (0.21–0.76)	2.09 (1.57–2.72)
*Hukou*	Rural	7,836	9.98 (9.21–10.66)	2.12 (1.81–2.46)	7.68 (7.10–8.29)	0.54 (0.39–0.72)	0.5 (0.35–0.68)	1.05 (0.83–1.30)	1.05 (0.83–1.30)	0.20 (0.12–0.33)	1.26 (1.03–1.54)
	Urban	3,253	17.83 (16.53–19.19)	4.21 (3.55–4.96)	10.45 (9.42–11.55)	1.57 (1.17–2.06)	1.44 (1.06–1.92)	5.66 (4.89–6.51)	3.93 (3.29–4.66)	1.48 (1.09–1.95)	3.72 (3.10–4.43)
Marital status	Married	4,896	12.01 (11.11–12.95)	2.08 (1.70–2.52)	8.31 (7.55–9.12)	0.88 (0.64–1.18)	0.71 (0.50–0.99)	2.45 (2.04–2.92)	1.84 (1.48–2.25)	0.61 (0.41–0.87)	1.98 (1.61–2.41)
	Others	6,193	12.5 (11.68–13.35)	3.25 (2.82–3.72)	8.64 (7.95–9.37)	0.81 (0.60–1.06)	0.82 (0.61–1.08)	2.36 (1.99–2.77)	1.94 (1.61–2.31)	0.55 (0.38–0.77)	1.99 (1.65–2.37)
Education level	Illiterate	5,224	10.16 (0.94–11.02)	2.32 (1.93–2.76)	7.68 (6.97–8.43)	0.52 (0.34–0.75)	0.52 (0.34–0.75)	1.19 (0.91–1.52)	1.15 (0.88–1.48)	0.31 (0.18–0.50)	1.42 (1.11–1.78)
	Primary and middle school	4,792	13.11 (12.16–14.09)	2.82 (2.37–3.33)	8.81 (8.02–9.64)	0.83 (0.60–1.13)	0.69 (0.47–0.97)	2.59 (2.16–3.08)	1.94 (1.57–2.37)	0.54 (0.35–0.79)	2.19 (1.80–2.65)
	High school and above	1,073	18.92 (16.62–21.39)	4.38 (3.24–5.78)	11.09 (9.27–13.12)	2.42 (1.59–3.53)	2.42 (1.59–3.53)	7.46 (5.96–9.19)	5.31 (4.05–6.83)	2.05 (1.29–3.09)	3.82 (2.76–5.15)
Living standard	Very good/good	2,168	18.68 (17.06–20.39)	4.75 (3.89–5.73)	12.32 (10.96–13.77)	1.80 (1.28–2.45)	1.71 (1.20–2.34)	4.61 (3.77–5.58)	3.64 (2.90–4.52)	1.29 (0.86–1.86)	2.95 (2.28–3.75)
	Fair	7,801	11.25 (10.56–11.98)	2.35 (2.02–2.71)	7.88 (7.30–8.50)	0.65 (0.49–0.86)	0.59 (0.43–0.79)	2.01 (1.71–2.35)	1.54 (1.28–1.84)	0.44 (0.30–0.61)	1.87 (1.58–2.20)
	Bad/very bad	1,120	7.05 (5.62–8.71)	1.52 (0.89–2.42)	5.36 (4.12–6.84)	0.27 (0.06–0.78)	0.27 (0.05–0.78)	0.80 (0.37–1.52)	0.98 (0.49–1.75)	0.18 (0.02–0.64)	0.89 (0.43–1.64)
Health status	Very good/good	5,203	12.68 (11.79–13.62)	2.94 (2.50–3.44)	8.76 (8.01–9.57)	1.00 (0.75–1.31)	1.00 (0.75–1.31)	2.52 (2.11–2.98)	2.00 (1.64–2.42)	0.69 (0.49–0.96)	1.92 (1.57–2.33)
	Fair	4,319	11.30 (10.37–12.28)	2.45 (2.01–2.96)	7.76 (6.98–8.59)	0.72 (0.49–1.02)	0.63 (0.41–0.91)	2.32 (1.89–2.81)	1.74 (1.37–2.17)	0.46 (0.28–0.71)	2.01 (1.62–2.48)
	Bad/very bad	1,567	13.66 (11.99–15.46)	2.81 (2.05–3.75)	9.64 (8.22–11.21)	0.64 (0.31–1.17)	0.45 (0.18–0.92)	2.23 (1.56–3.09)	1.98 (1.35–2.80)	0.51 (0.22–1.00)	2.11 (1.45–2.94)
Sleep quality	Very good/good	5,775	12.85 (12.00–13.74)	2.79 (2.38–3.25)	8.59 (7.88–9.34)	0.85 (0.63–1.12)	0.83 (0.61–1.10)	2.39 (2.01–2.82)	1.97 (1.63–2.37)	0.48 (0.32–0.70)	2.20 (1.84–2.61)
	Fair	3,616	11.06 (10.06–12.13)	2.43 (1.96–2.99)	8.21 (7.34–9.16)	0.80 (0.54–1.15)	0.69 (0.45–1.02)	2.41 (1.93–2.96)	1.77 (1.37–2.25)	0.66 (0.43–0.99)	1.69 (1.29–2.16)
	Bad/very bad	1,698	12.96 (11.39–14.65)	3.18 (2.40–4.13)	8.78 (7.47–10.22)	0.88 (0.50–1.45)	0.77 (0.41–1.31)	2.41 (1.74–3.26)	1.88 (1.29–2.65)	0.71 (0.37–0.12)	1.88 (1.29–2.65)
Smoke	Never	7,674	12.51 (11.78–13.27)	2.70 (2.35–3.08)	8.70 (8.08–9.36)	0.85 (0.65–1.08)	0.82 (0.63–1.05)	2.44 (2.10–2.81)	1.97 (1.67–2.30)	0.69 (0.52–0.90)	2.11 (1.81–2.46)
	Former	1,694	14.17 (12.54–15.92)	3.78 (2.92–4.80)	9.15 (7.82–10.62)	1.12 (0.68–1.75)	0.89 (0.50–1.46)	3.07 (2.30–4.01)	2.54 (1.84–3.40)	0.30 (0.10–0.69)	2.18 (1.54–3.00)
	Current	1,721	9.41 (8.07–10.89)	1.86 (1.28–2.61)	6.91 (5.76–8.22)	0.52 (0.24–0.99)	0.46 (0.20–0.91)	1.57 (1.04–2.27)	0.93 (0.53–1.51)	0.35 (0.13–0.76)	1.22 (0.76–1.86)
Drink	Never	8,104	12.64 (11.92–13.38)	2.81 (2.46–3.20)	8.65 (8.05–9.28)	0.80 (0.06–1.02)	0.72 (0.54–0.92)	2.44 (2.12–2.80)	1.92 (1.64–2.25)	0.58 (0.43–0.77)	2.07 (1.77–2.41)
	Former	1,320	12.20 (10.48–14.08)	3.56 (2.63–4.71)	8.03 (6.62–9.63)	1.44 (0.87–2.24)	1.29 (0.75–2.05)	2.58 (1.79–3.58)	2.27 (1.54–3.23)	0.38 (0.12–0.88)	2.12 (1.41–3.05)
	Current	1,665	10.63 (9.19–12.21)	1.68 (1.12–2.42)	8.11 (6.84–9.52)	0.54 (0.25–1.02)	0.66 (0.33–1.18)	2.04 (1.42–2.84)	1.44(0.93–2.14)	0.72 (0.37–1.26)	1.44 (0.93–2.14)
Exercise	Never	6,595	8.10 (7.45–8.78)	1.91 (1.59–2.27)	5.72 (5.17–6.30)	0.44 (0.30–0.63)	0.35 (0.22–0.52)	1.24 (0.99–1.54)	1.05 (0.08–1.32)	0.26 (0.15–0.41)	1.12 (0.88–1.41)
	Former	861	19.62 (17.03–22.44)	4.53 (3.24–6.14)	13.12 (10.94–15.56)	1.97 (1.15–3.14)	2.21 (1.33–3.42)	5.11 (3.74–6.80)	3.72 (2.56–5.21)	0.93 (0.40–1.82)	3.37 (2.27–4.80)
	Current	3,633	18.14 (16.90–19.43)	3.80 (3.20–4.47)	12.44 (11.39–13.56)	1.29 (0.95–1.72)	1.21 (0.88–1.62)	3.85 (3.25–4.53)	3.00 (2.47–3.61)	1.07 (0.76–1.46)	3.22 (2.67–3.85)

### Factors Associated With NS Use

Table 2 shows the results of the multivariate logistic regression examining the factors associated with NS use. The results displayed are for nine full models with all features entered into the logistic regression. Factors associated with the use of any NS included female gender, age 85–94, urban *hukou*, higher education, average and poor living standards, poor health status, former smoking, and exercise habits (former or current exercise). Factors associated with the use of protein included those aged over 85, urban *hukou*, higher education, average and poor living standard, former smoking, current drinking, and exercise habits (former or current exercise). Factors associated with the use of calcium included female gender, average and poor living standards, poor health status, former smoking, and exercise habits (former or current exercise). Factors associated with the use of iron included those aged over 75, high school education and above, average living standard, former smoking, and exercise habits (former or current exercise). Factors associated with the use of zinc included those aged over 85, high school education and above, average living standard, former drinking, and exercise habits (former or current exercise). Factors related to the use of multivitamins included female gender, those aged over 75, urban *hukou*, higher education, average and poor living standards, and exercise habits (former or current exercise). Factors related to the use of vitamins A and D included those aged 85–94 years, urban *hukou*, higher education, average and poor living standards, and exercise habits (former or current exercise). Factors related to DHA use included urban *hukou*, average living standard, average sleep quality, former smoking, and current exercise. Factors related to the use of other NSs included those aged over 85, urban *hukou*, higher education, and exercise habits (former or current exercise).

**Table 2:** Logistic regression analysis of factors associated with NSs use.

**Variables**		**Any NSs**		**Protein**		**Calcium**		**Iron**		**Zinc**		**Multivitamin**		**Vitamin A/D**		**DHA**		**Others**	
		**OR**	**95%CI**	**OR**	**95%CI**	**OR**	**95%CI**	**OR**	**95%CI**	**OR**	**95%CI**	**OR**	**95%CI**	**OR**	**95%CI**	**OR**	**95%CI**	**OR**	**95%CI**
Gender	Female	1.71^**^	1.46–2.00	1.25	0.92–1.70	1.79^**^	1.49–2.17	1.33	0.78–2.28	1.36	0.78–2.38	1.87^**^	1.35–2.60	1.36	0.95–1.96	0.69	0.37–1.27	1.35	0.94–1.92
Age,y	75–84	1.09	0.92–1.28	1.18	0.79–1.75	0.97	0.80–1.18	2.43^*^	1.18–5.00	1.84	0.90–3.79	1.59^*^	1.08–2.33	1.29	0.85–1.95	1.12	0.55–2.26	1.31	0.87–1.97
	85–94	1.30^**^	1.08–1.58	2.06^**^	1.37–3.08	1.08	0.87–1.35	3.85^**^	1.80–8.27	2.66^*^	1.23–5.74	2.34^**^	1.54–3.56	1.76^*^	1.12–2.76	1.26	0.58–2.75	1.87^**^	1.20–2.92
	≥95	1.18	0.95–1.46	2.70^**^	1.75–4.18	0.83	0.65–1.06	4.96^**^	2.14–11.51	3.05^**^	1.31–7.12	2.88^**^	1.80–4.61	1.46	0.86–2.47	0.91	0.35–2.37	2.03^**^	1.22–3.36
*Hukou*	Urban	1.25^**^	1.09–1.44	1.31^*^	1.00–1.72	0.95	0.80–1.12	1.29	0.78–2.12	1.19	0.71–2.00	2.86^**^	2.11–3.87	2.05^**^	1.48–2.86	3.95^**^	2.06–7.58	1.90^**^	1.39–2.59
Marital status	Others	1.04	0.90–1.21	1.23	0.91–1.66	1.09	0.92–1.29	0.68	0.41–1.14	0.96	0.56–1.65	0.88	0.64–1.20	1.14	0.81–1.60	1.27	0.69–2.34	0.91	0.65–1.27
Education level	Primary and middle school	1.32^**^	1.14–1.52	1.40^*^	1.04–1.88	1.14	0.96–1.35	1.68	0.95–2.95	1.40	0.78–2.52	2.16^**^	1.52–3.06	1.55^*^	1.06–2.26	1.09	0.54–2.20	1.46^*^	1.03–2.07
	High school and above	1.56^**^	1.25–1.94	1.70^*^	1.11–2.61	1.27	0.98–1.66	3.49^**^	1.72–7.05	3.66^**^	1.79–7.46	3.91^**^	2.55–5.99	2.80^**^	1.74–4.48	1.98	0.87–4.51	1.70^*^	1.05–2.74
Living standard	Fair	0.64^**^	0.56–0.73	0.59^**^	0.45–0.77	0.65^**^	0.55–0.76	0.54^**^	0.34–0.84	0.53^**^	0.33–0.84	0.66^**^	0.50–0.87	0.60^**^	0.44–0.81	0.54^*^	0.32–0.93	0.84	0.61–1.15
	Bad/very bad	0.42^**^	0.32–0.55	0.42^**^	0.25–0.73	0.44^**^	0.33–0.60	0.30^*^	0.09–1.01	0.35	0.10–1.19	0.40^*^	0.20–0.82	0.51^*^	0.26–0.99	0.34	0.08–1.50	0.50	0.25–1.01
Health status	Fair	1.02	0.89–1.17	0.96	0.73–1.25	1.00	0.85–1.17	0.78	0.48–1.24	0.70	0.43–1.15	1.02	0.77–1.35	0.99	0.72–1.36	0.73	0.41–1.31	1.26	0.93–1.71
	Bad/very bad	1.36^**^	1.13–1.63	1.10	0.76–1.59	1.35^**^	1.10–1.67	0.70	0.34–1.44	0.50	0.22–1.15	1.05	0.70–1.58	1.18	0.76–1.83	0.87	0.38–1.99	1.45	0.94–2.22
Sleep quality	Fair	0.92	0.81–1.06	1.00	0.76–1.32	1.03	0.88–1.21	1.21	0.75–1.96	1.12	0.68–1.86	1.19	0.89–1.58	1.02	0.74–1.41	1.84^*^	1.04–3.26	0.80	0.58–1.11
	Bad/very bad	1.01	0.85–1.21	1.29	0.93–1.80	1.01	0.82–1.24	1.30	0.70–2.39	1.18	0.62–2.26	1.09	0.75–1.58	0.99	0.65–1.50	1.88	0.92–3.88	0.83	0.55–1.25
Smoke	Former	1.33^**^	1.11–1.60	1.42^*^	1.01–2.00	1.28^*^	1.03–1.60	0.93	0.50–1.71	0.72	0.37–1.41	1.26	0.87–1.84	1.15	0.76–1.74	0.29^*^	0.11–0.77	0.96	0.63–1.47
	Current	0.96	0.78–1.18	0.92	0.61–1.41	0.99	0.78–1.26	0.70	0.32–1.52	0.58	0.26–1.31	0.97	0.61–1.53	0.58	0.33–1.03	0.47	0.18–1.18	0.71	0.43–1.19
Drink	Former	0.97	0.79–1.18	1.17	0.82–1.68	0.97	0.76–1.23	1.97^*^	1.09–3.56	2.32^**^	1.25–4.32	1.17	0.77–1.77	1.26	0.80–1.96	0.94	0.35–2.51	1.10	0.70–1.72
	Current	0.95	0.79–1.15	0.65^*^	0.42–0.99	1.11	0.89–1.37	0.78	0.37–1.65	1.20	0.59–2.41	1.04	0.69–1.56	0.92	0.58–1.48	1.50	0.73–3.05	0.84	0.53–1.34
Exercise	Former	2.24^**^	1.83–2.74	1.62^*^	1.10–2.38	2.43^**^	1.92–3.08	2.71^**^	1.43–5.14	4.21^**^	2.19–8.09	2.04^**^	1.37–3.03	2.08^**^	1.33–3.26	1.90	0.78–4.59	1.93^**^	1.22–3.05
	Current	2.28^**^	2.00–2.61	2.01^**^	1.53–2.63	2.87^**^	1.96–2.67	2.29^**^	1.38–3.82	2.70^**^	1.55–4.68	2.14^**^	1.58–2.90	2.07^**^	1.48–2.89	2.35^**^	1.26–4.39	2.51^**^	1.82–3.47

*Reference group in each variable is omitted. OR, odds ratio; CI, confidence interval*.

* *P < 0.05*,

** *P < 0.01*.

### Distribution of NSs Taken

**Figure 2:**
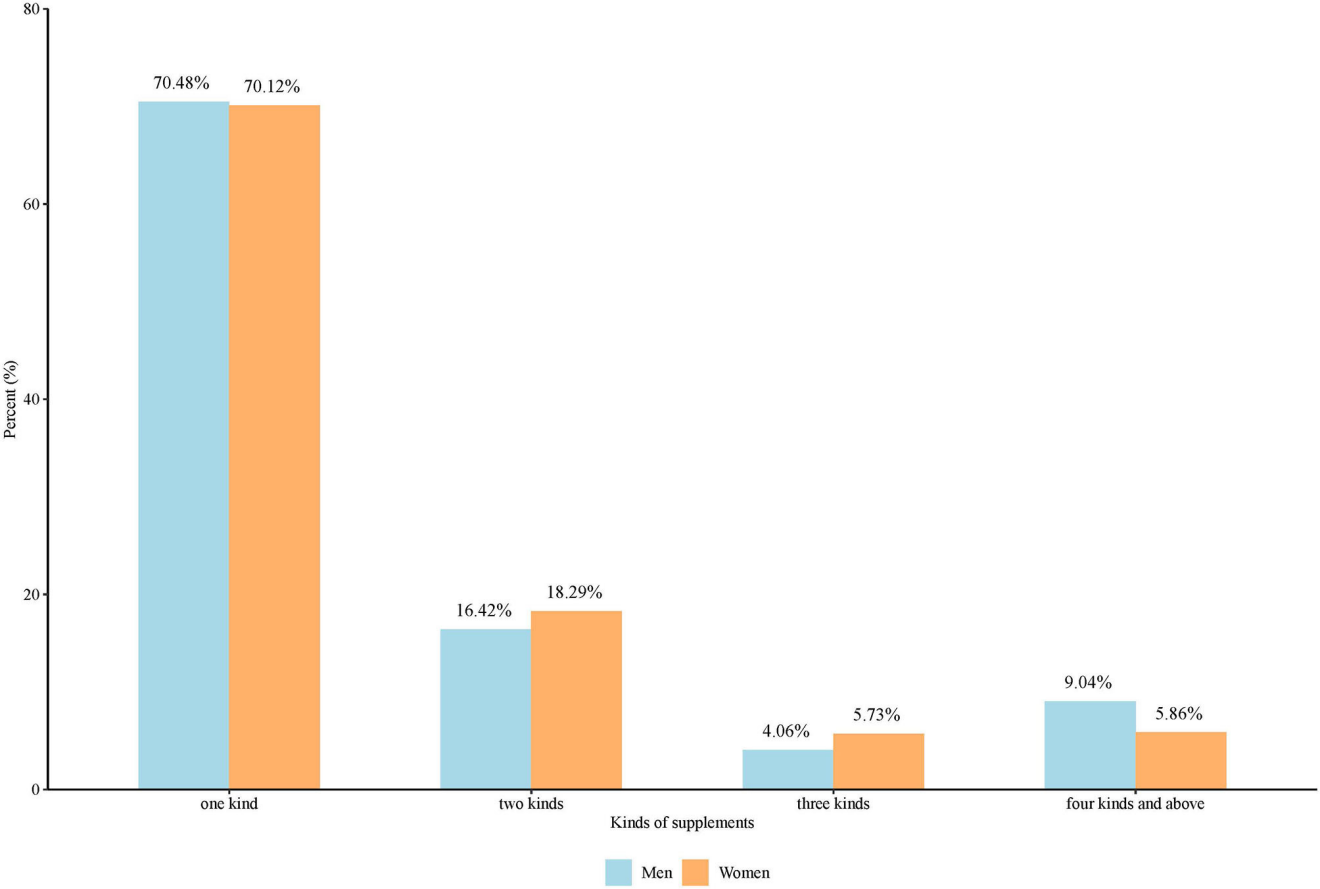
Distribution of the number of supplements taken by gender.

Figure 2 illustrates the distribution between men and women of the number of supplements taken, with 70.48% of males and 70.12% of females users taking only one NS; 9.04% of males and 5.86% of females took more than four or more NSs. There was a difference in the number of supplements taken by males and females, but the result was only statistically significant at the 10% level (χ^2^ = 7.113, *P* = 0.068).

**Figure 3:**
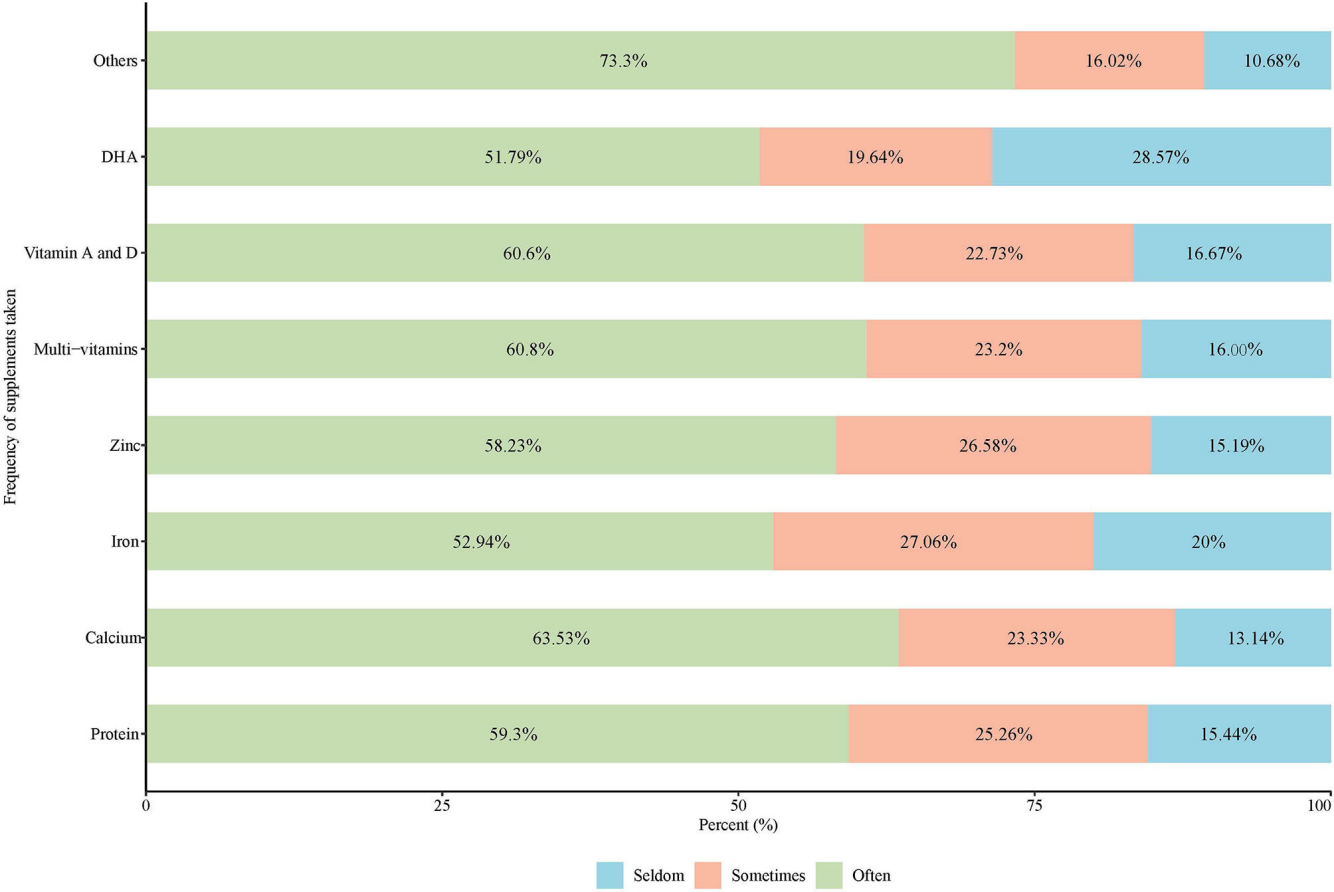
Distribution of the supplements taken by frequency.

More than 50% of NS users reported taking supplements often, with the top three most common types being others, calcium, and multivitamins. DHA and iron were used less frequently (Figure 3).

## Discussion

This study analyzed the use of NSs and associated factors among older Chinese adults aged 65 years and above. The results of the study showed that the probability of any NS use among older Chinese adults was 12.28%, significantly higher than the 1.75% among those aged 60 years and above reported by China Health and Nutrition Survey (CHNS) in 2010–2012(3) but still lower than the overall prevalence reported in developed countries, such as the United States, the NS use rate for those aged 60 years and above was 74.3% in 2017–2018(19). Previous studies reported that in Australian National Health Survey, the proportion of people aged 70 and over using NS was 52.7% in 2011–2012, and 49.5% in 2014–2015(20, 21). This may be because dietary guidelines in China prioritize adequate dietary sources of nutrients and do not recommend NSs for the general population (3, 22). The difference in prevalence is modest compared to older people in other Asian countries. South Korea reported 16.3% of the population aged 65 years and above using vitamin-mineral supplements in 2007–2008 (23), and Japan reported that the NS use proportion was 12.6% among males and 17.0% among females aged 70 years and above (24).

Our study showed that women used NSs, such as calcium and multivitamins, more often than men. This result is consistent with previous studies that have shown a higher interest in NS use among female consumers (25–27). Some researchers attribute this to women being more concerned than men about their health, as they feel more responsible for the wellbeing of other family members (2). It may also due to gender differences in genetic, epigenetic and hormonal mechanisms that lead to osteoporosis and iron deficiency anemia (IDA) being more prevalent in women (28–30). Older adults are more likely to use supplements such as protein, iron, zinc and multivitamins, and other studies have reported a significant linear increase in NS use in older age groups (24), which may be related to the increased health problems people face as they age. Higher levels of purchasing power and greater awareness of health care resulted in higher rates of NS use among urban than rural older adults (3). In addition, as in other studies, NSs were used more by older people who were well-educated and who had better living conditions (31, 32).

NS use also varied by health status and health awareness. This study showed that older people who rated themselves as having poor health were more likely to use NSs, which is consistent with the results of other Chinese studies (3, 33), but some researchers have reported opposite results, with a UK study finding that participants who considered their health to be good or excellent were more likely to use NSs than those who reported fair or poor health (34). As we mentioned earlier, the recommendations of the Chinese dietary guidelines possibly led older people who rated themselves as having good health to be more likely to obtain nutrients from their diet. The increased use of DHA among older adults with fair sleep quality compared to those with good sleep quality is because omega-3 polyunsaturated fatty acids, which include docosahexaenoic acid (DHA) and eicosapentaenoic acid (EPA), are beneficial for sleep (35). Several studies have shown that NS users tend to maintain a healthy lifestyle, such as a balanced diet and regular exercise (32, 34, 36). We found that former smokers were more likely to use NSs, including protein and calcium, and that former smokers increased supplement use, possibly to minimize the adverse effects of their previous habits on their health (27, 37). Past alcohol consumption was associated with the use of iron and zinc, and we suggest that this may be because most older people could not clearly distinguish between the effects of iron (a possible prooxidant) and zinc (a possible antioxidant) on cardiovascular disease and choose to avoid taking these supplements during periods of alcohol consumption (38). In addition, people with exercise habits, whether they currently exercise or not, had a higher probability of using a NS.

In terms of categories, the most common NS used by older Chinese people was calcium, followed by protein, which may be closely related to aging. The aging process is often characterized by unintended loss of muscle (sarcopenia) and bone (osteoporosis), and protein and calcium intake are factors often considered in the prevention or treatment of chronic osteoporosis and sarcopenia (39), with protein supplements and resistance exercise improving muscle function in frail older people (40). Due to differences in dietary habits, the Chinese choose to consume related nutrients more often through calcium supplements rather than in food (41). Apart from that, a large proportion of the Chinese population is at risk for inadequate intake of zinc, iron and vitamins (3), but this may be overlooked due to metabolic adjustments (42) and because there are no obvious clinical signs or symptoms of these deficiencies (43). Compared to other NSs, this study showed that the rate of iron and zinc intake in the elderly was <1%.

Notably, among respondents using NSs, the difference between men and women in terms of kinds of NSs taken was only statistically significant at the 10% level, with women preferring to take NSs in combination but controlling the overall quantity taken. In addition, those using NSs also showed a consistent pattern, with over 50% of supplement users reporting regular supplement use to maintain long-term organ-specific function (e.g., bone, heart, prostate protection) (44).

Our study analyzed the use of NS among older people aged 65 years and above in China. Although the results showed a relatively low rate of NS use in China, compared with rates reported in previous studies, the rate of NS use among older adults has increased, despite the different survey samples, since older adults are more likely to use supplements to maintain and promote health. However, as we have mentioned, inappropriate use of supplements may lead to adverse outcomes (44). In addition, with increasing health literacy, respondents expressed uncertainty about the usefulness of these supplements (45). Therefore, how do we provide scientifically based health guidance to older people that addresses their needs in the future? A recent systematic review showed that community pharmacists lack knowledge about NSs globally (46), suggesting that we should make NS-related health workers sufficiently knowledgeable about NSs to provide accurate information to users. At the same time, in the context of health policy and the development of general practitioners (GPs) in China, the management of people's health in the community by GPs should be strengthened, and personalized guidance should be provided to the elderly according to their health conditions and related needs, especially on the types of NSs to be taken and the frequency of taking them. As users of NSs are more likely to have a lower socioeconomic status, targeted NS guidance has benefits in reducing health inequalities and may also reduce adverse events due to inappropriate use (45).

Several potential limitations of this study should be noted. First, the cross-sectional design does not help us explore the trend of NS use among older adults in China. Although the database we used is a longitudinal survey, surveys of NSs started in 2018, and we hope in the future to be able to conduct tracking and comparative studies of trends in the use of NS among older people over time. In addition, due to the limitations of the data, our measurement of NSs is not comprehensive, and there is no information on specifications, quantities, etc., to help us calculate the intake of each nutrient. In particular, measures of NS utilization and self-rating questions such as living standards and health status were self-reported and could suffer from bias. Besides, factors including physical diseases, insurance, financial support need to be further explored in the future.

## Conclusion

The results of this study showed that the probability of using any NS among older Chinese adults was 12.28%. The most common NSs used by older Chinese individuals were calcium and protein. Women used NSs like calcium and multivitamins more often than men. Older people were more likely to use supplements like protein, iron, zinc and multivitamins. Older people with high levels of education, good living conditions and those who rated their health as poor were more likely to use NSs. Former smokers were more likely to use protein and calcium, past alcohol consumption was associated with iron and zinc use, and those with an exercise habit were more likely to use any of the NSs. More women reported taking 2–3 NSs, and more than 50% of NS users reported taking supplements often. This study provides information on the current prevalence of NS among older adults in China and the association with demographics, lifestyle and other factors. How to provide scientifically based health guidance on NS use for older people remains crucial in promoting their health in the future.

## Data Availability Statement

Publicly available datasets were analyzed in this study. The data of CLHLS 2018 is publicly available by application through http://chads.nsd.pku.edu.cn/sjzx/index.htm.

## Ethics Statement

The CLHLS study was approved by Research Ethics Committees of Peking University (IRB00001052–13074). The patients/participants provided their written informed consent to participate in this study.

## Author Contributions

WD performed and interpreted statistical analysis and drafted manuscript writing. ZS and RB supported manuscript writing and contributed to the study design for the study. All authors read and approved the final manuscript.

## Conflict of Interest

The authors declare that the research was conducted in the absence of any commercial or financial relationships that could be construed as a potential conflict of interest.

## Publisher's Note

All claims expressed in this article are solely those of the authors and do not necessarily represent those of their affiliated organizations, or those of the publisher, the editors and the reviewers. Any product that may be evaluated in this article, or claim that may be made by its manufacturer, is not guaranteed or endorsed by the publisher.
