# Supplementary material for: Prevalence of and Factors Associated With Nutritional Supplement Use Among Older Chinese Adults: A Nationwide Cross-Sectional Study in China
Source: Front Public Health. 2022 Mar 24;10:822087. doi: 10.3389/fpubh.2022.822087 (PMC8987002; doi:10.3389/fpubh.2022.822087)
Supplement: Supplementary file 1 [file Table_1.DOCX]

Supplementary Material

# Supplementary Tables

**Supplementary Table 1**. Questions and variables used in this study

| **Variable** | **Question** | **Classfication** |
| --- | --- | --- |
| Gender |  | Male; female |
| Age |  | 65-74, 75-84, 85-94, 95 years old and above |
| Hukou | Current type of household registration | Urban, rural |
| Marital status |  | Married, others (including divorced, widowed and never married) |
| Educational level |  | Illiterate, primary school and middle school, high school and above |
| Living standard | How do you rate your economic status compared with others in your local area? | Very good/good, fair, bad/very bad |
| Health status | The participant’s self-rated health | Very good/good, fair, bad/very bad |
| Sleep quality | Self-reported sleep quality | Very good/good, fair, bad/very bad |
| Smoke | Do you smoke regularly at present?"  "Did you smoke in the past? | Never, former, and current |
| Drink | Do you drink alcohol at present?"  "Did you drink alcohol in the past? | Never, former, and current |
| Exercise | Do you do exercises regularly at present?"  "Did you do exercises regularly in the past? | Never, former, and current |
| NS use | Do you usually take a nutritional supplement? | Yes/no |
|  | Do you take (1) protein, (2) calcium, (3) iron, (4) zinc, (5) multivitamin, (6) vitamin A/D, (7) docosahexaenoic acid (DHA) (8) others?” | Yes/no |
|  | “How often do you take each kind of nutritional supplement?” | Seldom/ sometimes/ often |
